# Planning an integrated agriculture and health program and designing its evaluation: Experience from Western Kenya

**DOI:** 10.1016/j.evalprogplan.2016.03.001

**Published:** 2016-06

**Authors:** Donald C. Cole, Carol Levin, Cornelia Loechl, Graham Thiele, Frederick Grant, Aimee Webb Girard, Kirimi Sindi, Jan Low

**Affiliations:** a Dalla Lana School of Public Health, University of Toronto, Toronto, Canada; b International Potato Center (CIP), Peru; c University of Washington, Seattle, USA; d International Atomic Energy Agency, Vienna, Austria; e CGIAR Research Program on Roots, Tubers and Bananas, Lima, Peru; f Rollins School of Public Health, Emory University, GA, USA; g CIP, Dar es Salaam, Tanzania; h CIP, Nairobi, Kenya

**Keywords:** Multi-sectoral program, Nutrition, Maternal and child health, Agriculture, Micronutrient, Vitamin A, Bio-fortification, Development evaluation

## Abstract

Multi-sectoral programs that involve stakeholders in agriculture, nutrition and health care are essential for responding to nutrition problems such as vitamin A deficiency among pregnant and lactating women and their infants in many poor areas of lower income countries. Yet planning such multi-sectoral programs and designing appropriate evaluations, to respond to different disciplinary cultures of evidence, remain a challenge. We describe the context, program development process, and evaluation design of the Mama SASHA project (Sweetpotato Action for Security and Health in Africa) which promoted production and consumption of a bio-fortified, orange-fleshed sweetpotato (OFSP). In planning the program we drew upon information from needs assessments, stakeholder consultations, and a first round of the implementation evaluation of a pilot project. The multi-disciplinary team worked with partner organizations to develop a program theory of change and an impact pathway which identified aspects of the program that would be monitored and established evaluation methods. Responding to the growing demand for greater rigour in impact evaluations, we carried out quasi-experimental allocation by health facility catchment area, repeat village surveys for assessment of change in intervention and control areas, and longitudinal tracking of individual mother-child pairs. Mid-course corrections in program implementation were informed by program monitoring, regular feedback from implementers and partners’ meetings. To assess economic efficiency and provide evidence for scaling we collected data on resources used and project expenses. Managing the multi-sectoral program and the mixed methods evaluation involved bargaining and trade-offs that were deemed essential to respond to the array of stakeholders, program funders and disciplines involved.

## Highlights

• Complex, integrated development programs present planning and evaluation challenges and opportunities.
• Multiple evaluation components are needed to respond to different disciplinary cultures of evidence in cross-sectoral programs.
• Delineating impact pathways helps visualize cross-sectoral outcomes and guides implementation monitoring.
• Feedback meetings are critical to build understanding across sectors and problem-solve in real time.

## 1 Introduction

In response to persistently poor maternal, newborn and child health indicators in low income countries (Countdown to 2015, 2016), governments, non-governmental organizations and international donors have intensified calls for multi-sectoral interventions. In 2013, the World Bank declared that “Nutrition is a multi-sectoral problem with multi-sectoral solutions” (World Bank, 2013). UNICEF also calls for multi-generational, multi-sectoral responses to these problems (UNICEF/EU, 2016). Given the vast majority of the rural poor globally are engaged in farming, promoting the cultivation and the consumption of micronutrient rich crops hold promise as complementary strategies to improve both livelihoods and nutritional status (Burchi, Fanzo, & Frison, 2011). However, as summarized in recent systematic reviews (Masset, Haddad, Cornelius, & Isaza-Castro, 2012; Ruel et al., 2013, Webb, 2013; Webb Girard, Self, McAuliffe, & Olude, 2012), the most appropriate multi-sectoral programs are not yet clear. Nor is there good evidence of the impact of such programs on the nutrition and health status of women and children, despite efforts to improve program evaluation (Levinson & Madzorera, 2005).

Although multi-sectoral interventions are increasingly espoused by development stakeholders, it is rare to see programs that involve agriculture, health and other relevant sectors., Like health promotion programs in other contexts (Jolley, 2014), operating at village, district and regional levels within low-income countries is intrinsically complex. Planning such programs involves interaction with multiple social actors and engaging in a range of activities in a development context where multiple goals are often of interest to funders and national governments (Bamberger, 2000). Program theory requires analyzing a complex chain of causality, often not adequately spelled out much less subject to rigorous evaluation (Forss, Marra, & Schwartz, 2011). Others who have evaluated complex rural development interventions (Luo & Liu, 2014) have also commented on these challenges.

Further, there are different traditions of evaluation rigour and cultures of evidence among those evaluating multi-sectoral interventions (Cole et al., 2003; Klein, 2008). Agricultural economists, nutritionists, human health researchers, development specialists and evaluators hotly debate what constitutes credible and actionable evidence to guide implementation and influence policy-making (Donaldson, 2015). Trickett et al. (2011) have argued that evaluations of community-level interventions require understanding diverse socio-ecological conditions across communities and collaborating with social actors at different levels and over time, in order to produce not only valid evidence but also sustainable community level impacts. Health promotion and implementation science researchers appeal for much more attention to how interventions are planned and implemented by partners across sectors and at multiple levels to promote synergies in impacts (MacLean et al., 2010). Those fostering innovation systems use participatory methods to map networks of social actors, identify and analyze scenarios, and model impact pathways for longer term uptake, and scaling up good ideas (Douthwaite et al., 2007). However, these more open-ended processes can result in more varied types and intensities of intervention, including “lapses, infidelities and creative adaptations” (Horton et al., 2013). These would likely be regarded as deficiencies among those who have called for greater rigour, championing randomized and quasi-experimental evaluation designs for a wide range of social, educational and economic interventions (see International Initiative for Impact Evaluation (www.3ieimpact.org/) and the Abdul Latif Jameel Poverty Action Lab at the Massachusetts Institute of Technology (http://www.povertyactionlab.org/about-j-pal)). In contrast, guidance on the evaluation of large scale development programs suggests paying attention to theories linking interventions to outcomes and advocates mixed methods approaches (Leeuw & Vaessen, 2009). There are practical problems for programs that must allocate resources to planning, implementation and evaluation, and may not have resources for a wide array of mixed-methods approaches (Morell, 2010).

**Fig. 1:**
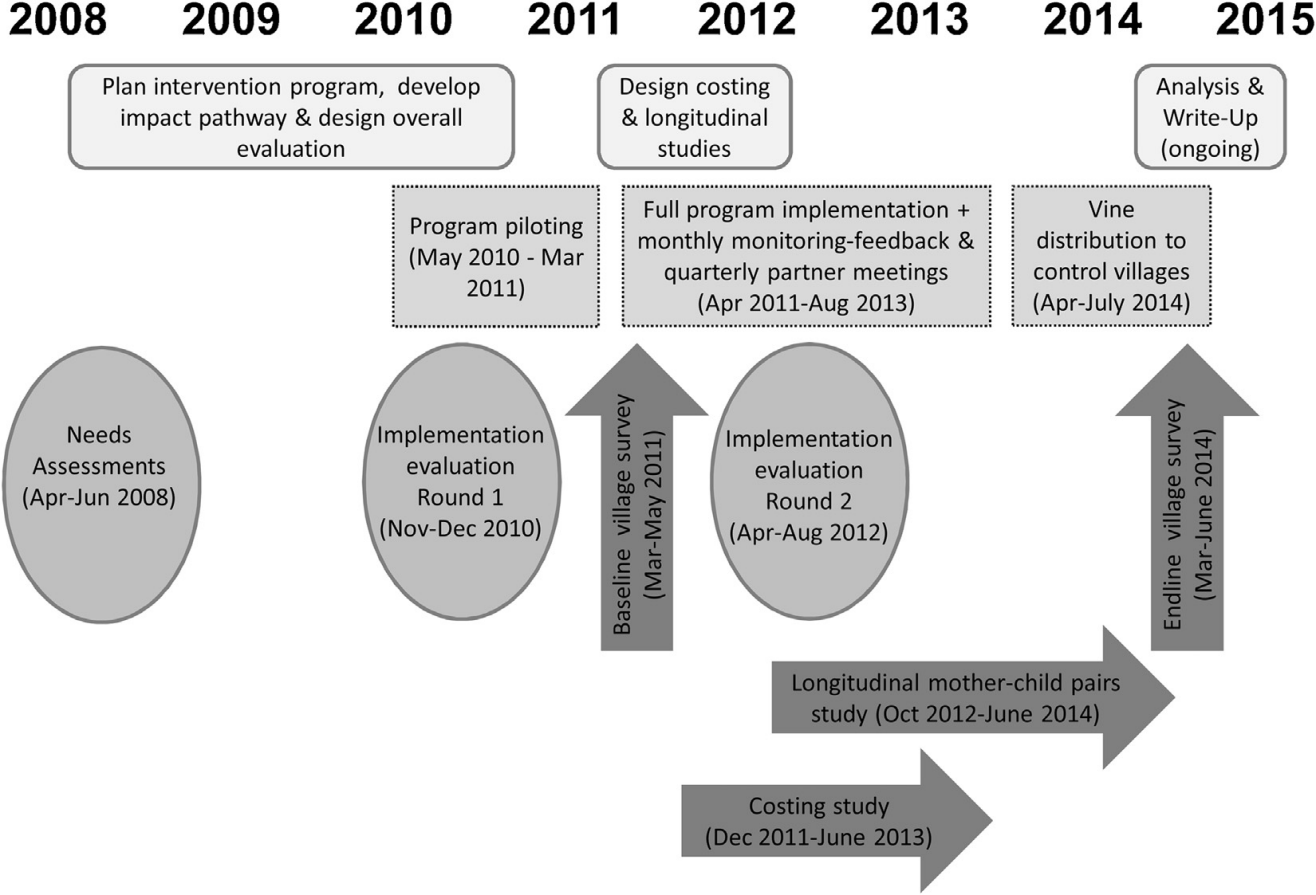
Mama SASHA planning, implementation and evaluation timeline.

As a multidisciplinary team involved in the planning of a multi-sectoral program and designing its evaluation, we faced both challenges in program planning and choices among the evaluation options, influenced by the priorities and approaches of our different disciplines. Our purposes in this paper are: to outline the multiple steps involved in planning the multi-sectoral program, including crucial linkage mechanisms across sectors; to describe our approach to monitoring and evaluation of its implementation; and to reflect on our experience of implementing the program and its evaluation (see Fig. 1). We start with the rationale for the program, its population focus, and the context. We next describe the needs assessment, planning with key social actors, and piloting of program components. All of these informed our program theory and helped us articulate expected pathways of change with the multiple interventions. To document implementation and outcomes, we formulated a mixed-methods, sequential design (Creswell & Plano Clark, 2010). We describe the benefits of our planning and evaluation approaches, the challenges and surprises we encountered along the way, and the adaptations required in both program implementation and our evaluation approach (Morell, 2010). Our aim is to share our experience of working across different cultures of evidence with other practitioners engaged in the planning and evaluation of new multi-sectoral programs in low income country contexts.

## 2 Rationale for the program

### 2.1 Internationally

Few well-designed studies have considered the interaction between agriculture interventions, nutrition education, health service utilization, and health and nutrition outcomes (Andrade et al., 2009; Webb Girard et al., 2012). Moreover, there has been limited study of the effects on pregnant and lactating women of multi-sectoral programs despite widespread recognition that “(p) regnancy to age 24 months is the critical window of opportunity for the delivery of nutrition interventions. If proper nutrition interventions are not delivered to children before the age of 24 months, [children] could suffer irreversible damage into their adult life…”(Ruel et al., 2013). Unfortunately, low uptake of antenatal and postnatal care and inconsistent nutritional information work against the improvement of nutrition practices for pregnant women and their infants.

In 2008, the International Potato Center (CIP),^1^ involved in agricultural research for development and the Programme on Appropriate Technology for Health (PATH), teamed up to add an agricultural component to an existing health intervention designed to improve nutrition outcomes. Earlier work (Hotz, Loechl, de Brauw et al., 2012, Hotz, Loechl, Lubowa et al., 2012, Jaarsveld et al., 2005, Low et al., 2007) had shown promise in reducing vitamin A deficiency by increasing consumption of the orange-fleshed sweet potato which was, however, not commonly available in the region of concern. The two organizations felt there was an opportunity to build on this research to link a nutrition-sensitive agricultural intervention more directly with health system efforts to prevent malnutrition and improve access to strengthened health services.

### 2.2 Nationally and regionally within Kenya

Though often under-reported in global health research, national and sub-national context is important for planning programs and interpreting results (Luoto, Shekelle, Maglione, Johnsen, & Perry, 2014). Kenya has struggled with persistently poor maternal and infant health statusand uneven linkages between the agriculture and health sectors (Alpha, 2013; IFPRI/FAO, 2013). The Western Province of Kenya became the geographic focus of our work. Similar to neighbouring Nyanza province, an estimated 23% of children aged 6–59 months were Vitamin A deficient in 2008 (Ruth et al., 2010). Coverage of Western province with Vitamin A supplementation to mothers and infants through the health system had generally been low − at approximately 15% to 40% depending on source (Clohossey et al., 2014), far less than the World Health Organization’s recommended 80% minimum coverage.^2^

Farming is the main economic activity and sweetpotato is an important staple food crop among smallholder farmers in Western Province. Grown predominantly by women, sweetpotato is consumed on a regular basis by young children and their mothers. However, the majority of sweetpotato varieties grown in Sub-Saharan Africa are either white- or yellow-fleshed, containing little or no beta-carotene. An agricultural challenge was to promote production of the improved beta-carotene–rich orange-fleshed varieties,^3^ drawing on CIP’s long-standing collaborations with the Kenyan Agricultural Research Institute.

Health services are provided by a mix of Kenyan government, private, and non-governmental organizations. NGOs are faith, humanitarian and/or development oriented with different national and international bases. PATH already had an existing relationship with the Ministry of Health through the AIDS, Population and Health Integrated Assistance Program (APHIA), funded by the United States Agency for International Development. APHIA aimed to improve comprehensive community and clinic based services, including maternal and antenatal health services, in areas with a high prevalence of HIV/AIDS such as Western Province.

## 3 Planning

### 3.1 Needs-Opportunity assessments

In 2008, CIP and PATH jointly conducted two needs and opportunity assessments. In April, we consulted with communities and their NGO partners, identifying nutrition training and promotion as wanting among agricultural extension agents, community health workers, and staff working in health facilities. When exploring the potential of OFSP to enhance nutritional intake, the team encountered perceptions of OFSP as “a women’s crop” and “a poor person’s food”. Nutrient rich foods were viewed as “sick person’s foods”, likely as a result of their provision to persons living with HIV or AIDS. Women of reproductive age and children less than five years of age were identified as important target groups for health and nutrition interventions because of their low immunity against infectious disease and their vulnerability to under-nutrition and vitamin A deficiency. Interviews with health-facility based workers in the first assessment revealed that women often came in late and infrequently during their pregnancy. Antenatal care services were falling short in effectively providing information and the means for pregnant women to actively improve their nutritional status during pregnancy.

A second two-week rapid appraisal in June focused on two counties—Bungoma and Busia. Key informant interviews and focus groups were held with facility and community-based health workers (facility and community), district nutritionists and agricultural extension agents. Even though they often felt overworked and underpaid, they were motivated by the satisfaction which came from serving their communities and working in an environment with effective supervision and cooperative teamwork. Health workers noted that multi-tasking and high client loads combined with the lack of adequate resources (staff, office space, materials) challenged their ability to effectively carry out their work. Ministry of Health nutritionists and Ministry of Agriculture home economists felt discouraged that nutrition programs and policies were under-financed, under-staffed and under-recognized in terms of their contribution to national development goals. They lacked training courses and access to current training materials.

In addition to these constraints, agricultural extension agents lacked transportation to reach farmers and perceived an unwillingness of farmers to adopt new crops and technologies. They noted that sweetpotato was viewed as a low-valued food crop, produced for children and women. Among community health workers and home-based care workers, the most frequently mentioned constraint to fulfilling their responsibilities was the lack of supplies and resources. Agricultural and health facility workers also mentioned that heavy service delivery workloads were compounded by a lack of high quality training to increase their effective knowledge when interacting with clients. Finally, both agricultural and health workers noted a tendency for projects to run in parallel leading to duplication of efforts and missed opportunities–reinforcing a need to integrate activities more carefully.

### 3.2 Program development

The needs assessments provided essential inputs to a January 2009 multi-stakeholder planning workshop. Included were scientists and practitioners from CIP, PATH, the Kenyan Agricultural Research Institute, NGO partners (the Appropriate Rural Development Agriculture Programme and the Community Research in Environment and Development Initiatives) and a representative of the Bill and Melinda Gates Foundation (BMGF), the potential funder. In keeping with joint planning approaches to evaluation (Sridharan & Nakaima, 2011), we talked through the needs and opportunities, both scientifically and practically on the ground. We sketched out an initial program theory, clarified objectives, laid out components, set initial targets, brainstormed evaluation approaches, and elaborated an initial concept note. The latter became part of the first phase of SASHA (Sweetpotato Action for Security and Health in Africa) − a five year effort led by CIP and financed by the BMGF. Although the BMGF’s Agricultural Development team was the lead, their Global Health team members also reviewed our concept note with their own criteria, requiring additional clarifications. Hence several rounds of revision between CIP, PATH and the Foundation were required. Nevertheless, the BMGF funded the Western Kenya proof-of-concept sub-component of SASHA, known as Mama SASHA, in the third quarter of 2009.

### 3.3 Goal and objectives

Mama SASHA’s overall goal was to improve the health status of pregnant women and the nutritional status of children up to two years through an integrated OFSP and health service-delivery strategy in Bungoma and Busia counties of Western Kenya. To meet this goal, the team set out three broad objectives:

1. To strengthen aspects of existing information, education, and communication materials and methods for supporting sustainable OFSP production and consumption at both the health facility and community levels, i.e. to improve the knowledge and practices of health workers, agricultural extension agents, and community members about OFSP and vitamin A rich foods.
2. To improve the evidence base on impacts (on nutrition, women and child health status, and use of health services) and on sustainability of a delivery system for high-yielding OFSP through community- and facility-based health services, in conjunction with agricultural partners.
3. To understand the costs and benefits of linking an integrated OFSP-focused agricultural-nutritional intervention to a health service-delivery system serving pregnant women.

### 3.4 Program activities

Prior to program initiation, the agricultural team conducted an agronomic and consumer acceptance study of potential OFSP varieties, in order to select two for use in Mama SASHA. Each of the national agricultural NGO partners worked with seven vine multipliers located near to each health facility and associated villages, who established and maintained OFSP vines for distribution. Vine multipliers were provided with a flip chart that described available OFSP varieties, how to select and maintain quality planting material and how to avoid infestation with the sweetpotato weevil, a major pest. They were also trained to provide information on nutritional benefits of OFSP, including vitamin A. The benefits of producing and consuming OFSP were conveyed to the larger community through semi-annual field days that highlighted the new varieties and methods for preparing OFSP.

Existing community health workers encouraged pregnant women to seek recommended early antenatal care (ANC) and postnatal care services. Mama SASHA trained and supported community health workers to implement community-level clubs for pregnant and lactating women, with monthly dialogue sessions on nutrition and health topics, and cooking demonstrations that utilized OFSP and other vitamin A rich foods. They used information, education and communication materials developed as part of Mama SASHA including a manual for conducting monthly club sessions. Large posters at each participating health facility encouraged women to come early in the pregnancy to ANC services.

During each health facility visit, nurses and/or community health workers provided improved maternal, infant and pre-school child nutrition counselling.^4^ Pamphlets with key messages on healthy eating were produced for nurses to give to the pregnant women attending antenatal care sessions. The major communication aid for facility based nurses was a desk-sized set of counselling cards with six lessons developed by project members and PATH practitioners and extensively pre-tested. Each page on the chart had illustrated examples of healthy practices on the front, with the accompanying messages on the back (4–5 key messages per topic). The major lessons were: 1) Healthy mothers during pregnancy; 2) Healthy eating; 3) Vitamin A; 4) Infant Feeding; 5) Orange-fleshed Sweetpotato Benefits; and 6) Growing Orange-fleshed Sweetpotato. At each ANC visit, the nurse or community health worker was to conduct lesson #5 and present one additional topic.

Crucially, nurses also dispensed vouchers for OFSP vines, thus linking to the agricultural side of the program. The voucher innovation was modelled on other successful uses of vouchers to promote use of services in reproductive health (Bellow et al., 2013; Janisch, Albrecht, Wolfschuetz, Kundu, & Klein, 2010), maternal and child health^5^ and agriculture (Carter, Laajaj, & Yang, 2013). However, using the vouchers in the health sector to stimulate demand for vitamin A rich OFSP, and coordinating that with its supply via OFSP vines through the agriculture sector was novel. Pregnant or lactating women received two vouchers per visit to the health facility, which they (or their family members) could redeem for 100 fresh cuttings each of improved Kabode and Vita varieties of OFSP from the vine multipliers. Trained agriculture extension officers from the Ministry of Agriculture extension services, NGOs and CIP followed up with agronomic advice and home visits to assess and discuss OFSP planting and crop management, providing another agriculture-health linkage.

Given the novelty of the cross-sectoral approach, the Mama SASHA team and partners were sufficiently uncertain about the program components and their linkages that a pilot was undertaken (2010) in sub-locations outside those intended for full implementation. To assess the acceptability and feasibility of introducing of OFSP and nutrition related activities,^6^ we conducted implementation evaluation (i) through key informant interviews and focus group discussions. Given our needs assessment findings, we were particularly interested in the impact of the additional activities on workload and resources in the health system, the agriculture system and households participating in the project.

The OFSP related activities were found to be worthwhile and acceptable to health, agriculture and community actors and the beneficiaries. Evidence that more women were taking up ANC services in the pilot area, many for the first time, was encouraging. Planting and consumption of OFSP was increasing, gradually replacing the traditional white and yellow sweetpotato varieties. A key set of recommendations focused on strengthening male partner involvement, in addition to male community leaders, as they could play either a powerful blocking or enabling role in allocating sufficient land for OFSP, providing agricultural labour and emotional support during difficult periods of a woman’s pregnancy, purchasing other nutritious foods in the market, and promoting consumption of OFSP in the home.

Informants and focus groups made several suggestions for strengthening the integrated agriculture, nutrition and health activities, including; additional training of implementing staff; development of more education materials for use by implementers as well as participating women; standardization of voucher provision and agronomic and nutrition activities; simplification of some monitoring tools (see Monitoring below), and the support of an integrated data management system in keeping with good practice in international development (Guerra-López & Hicks, 2015).

Integration across multiple sectors required substantial coordination, networking and organizational support with relevant social actors at different jurisdictional levels. We conducted awareness work with chiefs and community leaders at the village or sub-location level; discussions with agricultural, nutrition and health service managers at the location and county level; and initial and repeated joint training of agriculture, nutrition and health staff working with Mama SASHA. We also held regular meetings including monthly feedback to agriculture, nutrition and health staff and community partners within a given facility catchment area quarterly meetings with implementation partners; regular teleconferences with the research-evaluation team; annual sessions with the broader network of stakeholders in Western province; and annual funder visits. Table 1 provides a summary of these critical organizational meetings, which promoted integration throughout the program.

## 4 Program theory

The needs assessments, earlier literature (Andrade et al., 2009), program planning, pilot implementation evaluation and prior work delineating pathways of change in agriculture-health promotion work (Orozco, Cole, Ibrahim, & Wanigaratne, 2011) all informed Mama SASHA’s program theory. We undertook a more explicit and shared examination of potential impact pathways through a participatory impact pathway analysis (Alvarez et al., 2010) with key stakeholders and implementation partners at a two day workshop in 2010.

### 4.1 Impact pathway

**Fig. 2:**
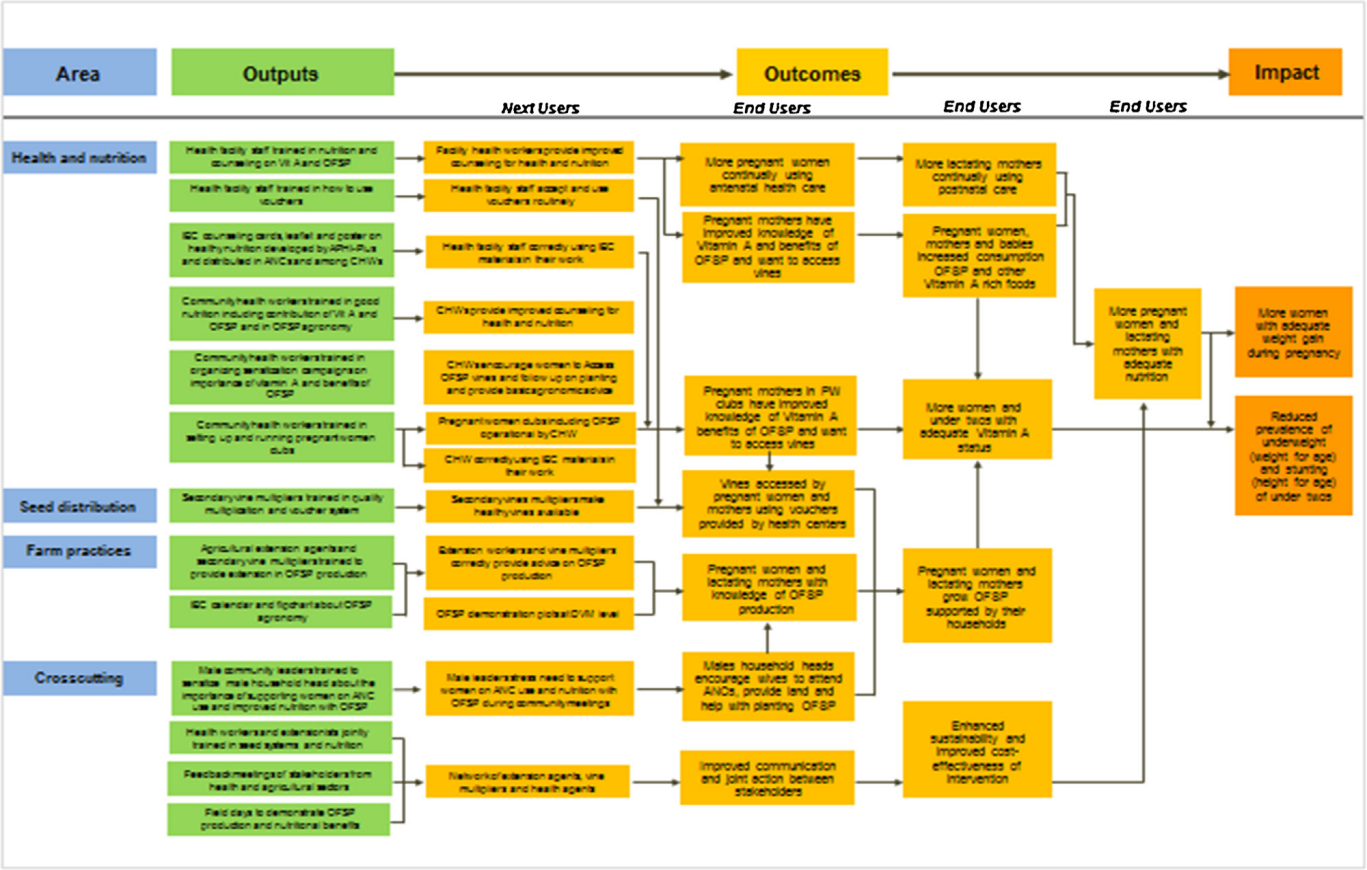
Mama SASHA impact pathway.

The overall impact pathway included all major areas of action (1st column, Fig. 2): health and nutrition, seed systems, farm practices and cross-sectoral integration. Moving left to right, the Impact Pathway described the underlying theory of change linking project outputs (2nd column) with outcomes among next users. The latter were part of the implementation team (3rd column) who promoted outcomes among the end users, referred to by some as the target population or by funders as beneficiaries (4th-6th columns). Impacts can be related to important national and global development goals. For example, trained health facility staff and community health workers were expected to use educational materials for counselling, to improve women’s knowledge of OFSP and intake of Vitamin A rich foods more generally. These results would in turn be associated with improved Vitamin A status and reduced underweight and stunting among children less than two years of age. A key link between these and the parallel vine multiplication and on-farm areas of activity were the vouchers for vines and the gender-related work with male community leaders and household heads. As in other health promotion programs (MacLean et al., 2010), considerable effort was required to foster integration across sector and along chains of responsibility. Integration was a key focus of the regular Mama SASHA meetings (Table 1), with ongoing reference back to the impact pathway to remind all involved of their respective contributions to achieving the goals of the multi-sectoral interventions.

### 4.2 Monitoring

Similar to other approaches for documenting implementation (Aarestrup, Jørgensen, Due, & Krølner, 2014), the impact pathway became the basis for setting up an integrated monitoring system of activities. Each output and outcome on the impact pathway required detailed consideration of which variables to measure, when and how to measure them, and who was responsible (full set of monitoring forms available upon request). For example, the monitoring of health activities included tracking club sessions for pregnant women, antenatal and postnatal care facility visits, and women receiving vouchers at such visits. Experience in the pilot led us to simplify health activity monitoring forms to reflect feedback from the nurses and community health workers. On the agriculture side, we tracked the redemption of vouchers for vines, visited a random selection of homes to ascertain whether and how vines were planted, and recorded monitoring visits by agricultural extension officers. We also conducted assessments of relevant early (or proximal, to use Aarestrup and colleagues’ term) end-user outcomes such as production knowledge and consumption of OFSP. A food frequency tool, applied monthly in different sites on a sample of participants, gave us indications of changes in these OFSP indicators during implementation.

Such measures provided valuable information on differential implementation progress across intervention facilities and their associated sub-locations, the units of analysis in our controlled design (Hawe, Shiell, Riley, & Gold, 2004). They also provided key inputs for discussion at monthly feedback meetings, lasting 2–4 h at each intervention health facility (see above and Table 1). All key actors on the ground (vine multipliers, nurses, community health workers, agriculture extension personnel) attended these meetings, which were facilitated by the PATH project officer. Actors both provided their data to the project monitoring staff and, through group discussions enabled timely detection of problems in implementation and monitoring in keeping with participatory monitoring approaches (Guerra-López & Hicks, 2015). Together, they worked on problem resolution − for example, when it was noted that project participants from one set of villages were not collecting vines, actors in the field provided the opportunity to collect them from a vine multiplier closer by road transport to their villages, despite being formally assigned to another intervention facility. Similarly, some health facilities were short of staff and nurses found it hard to carry out one-on-one counselling and filling in forms. An adaptation was for selected community health workers to work with nurses on group education activities during ANC clinics, as an alternative form of facility-based education.

## 5 Mixed methods evaluation approach

For the program’s major funder, Mama SASHA’s evaluation corresponded to “an evaluation to test the causal effects of pilot projects, innovations, or delivery models” (BMGF, 2016). The Mama SASHA evaluation design team included agricultural economists, nutritionists, a physician-epidemiologist and an anthropologist, each with their own traditions in evaluation. Guidance by Levinson and Madzorera (2005) and Habicht, Pelto, and Lapp (2009) was helpful in informing our initial evaluation design, and our approach was supported by later guidelines for evaluations of agriculture and health intervention programs (Ruel et al., 2013, Webb, 2013). The last argued for evaluations to: 1) explicitly hypothesize pathways of action through the use of program theory and impact pathway assessments; 2) be methodologically rigorous both in terms of quantitative and qualitative assessment so that programs adequately assess the determinants of differential program participation as well as the effects of such participation; 3) include a range of effect indicators; and 4) assess cost, cost-effectiveness and scalability. Given the multiple disciplines in the evaluation team, we chose a multi-method evaluation approach, which is strongly supported in development evaluation guidelines (Leeuw & Vaessen, 2009) (see Table 2 for components). We used our impact pathway to “leverage integration” (Klein, 2008), similar to the use of program logic models as an integrative framework (Cooksy, Gill, & Kelly, 2011) in other evaluations of complex program interventions.

As outlined in Table 2, we started with a baseline survey of intervention and control villages in March-May 2011, followed immediately by voucher distribution in intervention areas. Shortly thereafter, a costing study was designed using monitoring data. In mid-2012, we contracted a second round of implementation evaluation, using the same consultancy for consistency. Design work, funding applications and research ethics reviews for a longitudinal study of mother-infant pairs occurred concurrently, with a start in November 2012. We completed an endline survey in intervention and control villages in March-June 2014, with the timing chosen to match the earlier baseline survey. Each component of the project underwent ethical approval from KEMRI (Kenyan Medical Research Institute) and at least one of PATH, University of Toronto and/or Emory University.

### 5.1 Randomized allocation of program interventions

Keeping in mind the general call for more rigorous study designs, with counterfactuals (see Introduction and Leeuw & Vaessen, 2009), we aimed for comparisons between villages clustered around health facilities which initially would and would not receive the program. Eight facilities and villages from Bungoma and Busia counties were selected according to size-related variables (number of service providers, facility attendance numbers, and population served), coverage with community health workers linked to APHIA and location criteria. The latter included separation of facilities by approximately 30–50 km to reduce contamination. The selected health facilities and their associated villages (approximately 10–15/facility depending on village and catchment size) were then randomly assigned to intervention or control for the implementation period. The four intervention facilities and their associated villages were involved in the full range of agricultural, nutrition, and health service program activities, as described above, from the beginning of full implementation. The four control facilities continued to receive the standard APHIA training and sensitization on Infant and Young Child Nutrition services, but without the pregnant women’s groups, vouchers, or agricultural support for the production of OFSP. Some enthusiastic agricultural extension officers supplied OFSP vines to farmers close to one of the control areas, but uptake of these vines in the control villages was minimal. In keeping with ethics review commitments, these control facilities and villages received vines after the endline survey.

### 5.2 Implementation evaluation (ii)

The second round of implementation evaluation aimed to check on continued acceptability and emerging benefits, to explore the reasons why some women were not redeeming their vouchers, and to provide feedback to implementers. Like round 1, the evaluation used key informant interviews and focus group discussions.^7^ All implementing actors were motivated by enhanced training, more effective nutrition messaging, tangible recommendations to improve dietary quality, community recognition, and ability to better serve their clients. Emerging benefits included participant perceptions’ of enhanced maternal and child health coupled with greater food security. Mothers felt their children were less susceptible to disease and more energetic; they and their male partners also valued OFSPs’ shorter time to maturity and higher yields. Frontline health workers perceived higher antenatal care attendance and increased contact with mothers, their partners and children; only a few were concerned by the heavier workload.

Community health workers emerged as key facilitators of implementation and outreach to mothers. They and the vine multipliers also noted the challenge of increased workload without commensurate remuneration. Community health workers received a small stipend monthly for transport costs, but no salary. Initially, this stipend was 1000 Kenyan shillings (Ksh)(approximately $12.5 USD) per month, which was paid by the APHIA project. During the last year of the intervention, this stipend was reduced to 500 Ksh due to the Ministry of Health’s need for cost savings. This demoralized many community health workers and led to significant performance reduction among some of them. In addition, some mothers faced long distances in travelling to facilities for antenatal care and to vine mulitiplier locations for vines. Some participants feared that OFSP had contraceptive properties, a myth that was counteracted with the help of community leaders and in monthly dialogue sessions.

Some women felt that they only needed to redeem 2 of the 4 vouchers offered as those would provide sufficient planting material. This is because women’s plot sizes were generally small, ranging in size from 6 by 2–20 by 2 m, and once they received vouchers, women were able to multiply the planting material as needed. A major factor influencing voucher redemption was whether men in the household would allocate land to women for OFSP production. As a response, additional sessions were held to sensitize male leaders in intervention villages about the importance of OFSP production, and to urge them to convince other men in their village about the role of OFSP in promoting the health of their women partners and their children. This strategy proved effective in increasing area allocated to OFSP.

### 5.3 Repeat household surveys

We designed a survey for the intervention and control villages to provide comprehensive, and representative baseline data on OFSP knowledge, farming and consumption in the target area as well as health and nutrition status of pregnant women, children aged 6–23 months and their mothers. Survey modules queried household characteristics, food security, dietary diversity, health and nutrition knowledge, attitudes and practices, health service utilization, and agricultural production activities including of OFSP. We also collected anthropometry data and blood for retinol binding protein, a biological marker of Vitamin A status. A complementary endline survey conducted in the same villages (with adjustment for administrative changes in village definition over the period), included the same kinds of populations (with partial repeat participation for women with new pregnancies or young infants) and measurement domains. We added questions on degree of exposure to Mama SASHA interventions and extent of participation in project activities among intervention village households, a key way of documenting co-interventions.^8^ With these we aimed to assess changes in community prevalence, means or medians over the three years, comparing the intervention versus the control areas.

Although pre-post assessments with a co-temporaneous counterfactual added to rigour, they required substantial resources, with cost over-runs each time. A large survey team worked for several months each round in order to complete two censuses prior to the survey, to document which houses included pregnant women and women with young infants. Ensuring adequate response rates in rural areas required a substantial organizational and logistic effort, including provision of transportation. Both the baseline and endline were conducted from March-May, a period of time known as the hunger season because stocks of maize, the major staple in the area, are often depleted and it is planting time, requiring significant energy outlays. Thus, we were assessing change in multiple outcomes (OFSP knowledge, production, and consumption and antenatal care and post-natal care utilization) and impact (child underweight and stunting), at the most challenging time of year for participating households.

### 5.4 Longitudinal study of mother-infant pairs

In order to track change among pregnant women and then the mother-infant pairs, we focused on a smaller population whom we followed over time. We were interested in not only in how uptake of OFSP interventions would affect nutrition, health services utilization and health status of mothers and their infants, but also biological indicators of micronutrient status (Grant et al., 2014)^9^ rare among intervention studies (Hotz, Loechl, de Brauw et al., 2012, Hotz, Loechl, Lubowa et al., 2012, Low et al., 2007) but essential to meet expectations of the nutrition community. This component enrolled pregnant women in mid pregnancy (10–24 weeks) from six of the same facilities (4 intervention, 2 control) for the Mama SASHA intervention and two new control facilities in the same districts. The latter was due to concerns that other projects using OFSP might be distributing in the two remaining Mama SASHA control areas. We aimed to follow these women and their infants from enrollment through 10 months postpartum, to a 4th and final visit. We planned on 10% lost to follow-up but over 20% ceased participating. Attrition was due to miscarriages or stillbirths suffered and moving out of the area for their delivery or early post-partum to be with family members in other geographical areas. Tracking participants over time was staff intensive.

The key outcomes for this longitudinal study were 1) vitamin A status of mothers (during and post-pregnancy), measured as breastmilk retinol and serum retinol binding protein (RBP) adjusted for inflammation (C-reactive protein and alpha(1)-acid glycoprotein); 2) vitamin A status of infants (at 4 and 9 months), measured as serum RBP adjusted for inflammation; 3) hemoglobin concentrations of mothers and their infants measured using Hemocue and 4) anthropometry of mothers and infants. To improve nutrition intake measurement precision, a subsample of women and their infants were also recruited at 9 months postpartum and participated in multiple-pass 24-hour dietary recalls to quantify vitamin A and other nutrient intakes. Such biological measurement required substantial infrastructure investments in the selected health facilities (solar electricity generator, freezers, centrifuges and the like) and training of dedicated assistants based at those facilities.

### 5.5 Costing

Given the kinds of trade-offs and choices which policy makers face in achieving development goals and the need to speak to both agricultural and health economics’ communities, we sought to provide a detailed analysis of resource use and financial costs of establishing and running the three-year multi-sectoral intervention program. Similar to costing done for the HarvestPlus Reaching and Engaging End Users Project (HarvestPlus 2010),^10^ we estimated resource use across all participating sectors from a provider perspective. We estimated both financial and economic costs, with the latter capturing the opportunity cost of all resources (i.e. labour) used, regardless of whether the project paid for it or not. We excluded evaluation costs, as we were interested more in what would apply post project when sustainability and scaling up were the overarching context for resource use decisions. We sought estimates of total costs, costs per beneficiary and cost per contact and detailed profiles of cost shares by activities and inputs.

Using an expenditure approach, we captured the financial costs associated with planning, training, materials development and delivery of agriculture, nutrition and health support services, supplies and equipment.^11^ To better understand personnel time allocated to specific Mama SASHA activities, in 2013 we conducted focus group discussions and key informant interviews with agricultural extension officers, NGO partners, facility based health workers, and community health workers. We also used these meetings to clarify activity and input codes. We adopted an incremental rather than total resource use approach for those activities within the purview of the Ministries of Agriculture and Health. For example, we estimated the incremental financial costs for the existing antenatal and postnatal care at health facilities and for additional supports to community based health activities, where APHIA had already established cadres of community health workers. However, NGO activities were new, so costs for managing the receipt and redemption of vouchers, as well as initiating and supervising agricultural activities for establishing vine multiplication of new varieties of OFSP were all included.

The main difference between the financial and economic cost analyses was that the economic analysis had to value all personnel time used to deliver the intervention program by collaborating partners and included beneficiary time to participate. In order to capture economic costs, we conducted focus group discussions and semi-structured interviews on time allocation from shared personnel from the Ministries of Health and Agriculture, volunteer labour from the community, and the beneficiary labour for participating in the program. To estimate the full economic costs, we added the opportunity cost of time to the financial costs.

### 5.6 Cost-effectiveness analyses

Cost-effectiveness analysis is sought after by health policy makers to inform intervention prioritization and generate an evidence base to support scaling (see the World Health Organization’s site on Cost Effectiveness and Strategic Planning www.who.int/choice/en/). Typically, the analysis incorporates the opportunity costs of all resources used to delivery health and agriculture services to beneficiaries, while capturing both the health and economic benefits associated with participation in the intervention. For Mama SASHA, the cost-effectiveness analysis used the economic cost data along with health outcome data on vitamin A status, anemia, and nutritional status data from both the repeat surveys and longitudinal studies. Economic benefits captured primarily fell into two categories: value of OFSP production for vine multipliers and beneficiaries, and treatment costs averted.

Cost-effectiveness analysis (CEA) relates the net costs associated with a health outcome, such as cost per disease avoided, cost per death avoided, or cost per additional expected life year. However, integrated nutrition-sensitive strategies are designed to meet several nutrition and other desired outcomes, so have multiple inter-related outcomes of interest. Disability adjusted life years (DALYs) are a standard metric that summarizes morbidity and mortality burden attributable to a specific disease (Drummond, Sculpher, Torrance, O’Brien, & Stoddart, 2005). Because the intervention was hypothesized to have multiple nutrition and health benefits, DALYs were calculated from a whole program perspective. Even then, the estimate DALYs averted did not capture the full range of benefits and outcomes resulting from a multi-sectoral approach. These would include improved household food security, the long-term benefits of improved r sweetpotato knowledge, and future income earnings form OFSP production and sales of excess production, once household needs were met.

## 6 Reflections on planning and evaluation design

Our approach to planning and evaluation of a multi-sectoral intervention program in a rural setting of a low income country drew on a wealth of options we have noted throughout this paper. Overall, we were surprised at how long the planning process–needs assessments, program development, intervention activities piloting, implementation evaluation, and impact pathway development took–years versus the usual months allocated by most funders. We were fortunate to build on the core resources of CIP and PATH, to have donated time by university partners, and to have a funder who understood the importance of refining the multi-sectoral program. Further, the funder, stakeholders and team were supportive of mixed methods evaluation, including additional evaluation resources beyond those available to many programs. Another example of substantial evaluation resources for a multi-sectoral intervention program was the Millennium Villages Project (2016) (Pronyk et al., 2012). This funding context for adequate planning and evaluation is unfortunately less common in the competitive consultancy or peer review grants field than we think is helpful for innovative programs.

The need to respect traditional approaches to rigour, common in the agricultural and health sciences (Webb & Kennedy, 2014), all placed demands upon the evaluation team and program staff which expanded the scope of our evaluation. Our decisions resonate with the choices facing other colleagues evaluating multi-sectoral programs. Menon, Rawat, and Ruel (2013) clearly lay out the challenges in thinking through the feasibility of contextually relevant designs in evaluation of complex interventions in low income countries, when a key demand is for rigorous impact evaluation evidence. They argued strongly for early “implementer-evaluator engagement”, something which we realized required substantial time and interaction during the planning phase. Menon and colleagues also included a theory-driven process evaluation component, primarily to study factors that facilitate or prevent achieving impact, rather like our implementation evaluations. Our impact pathway was used to define monitoring and evaluation indicators and formed the basis for regular checks on progress by implementing partners, stakeholders and evaluators. In retrospect, we realize it could have been updated more fully over time to reflect and capture subtle changes in program implementation, user feedback, and un-expected outcomes.

Olney, Talukder, Iannotti, Ruel, and Quinn (2009) initially found the use of a classic pre-post design to be inadequate to the evaluation task of demonstrating the full range of expected outcomes of a homestead food production program in Cambodia. Retrospectively, they worked with the implementing organization to elucidate a program theory and impact pathways. They used qualitative methods to pinpoint gaps in implementation which might account for breaks in the causal linkages posited in the impact pathways (Olney et al., 2013). Our implementation evaluation (i) successfully fulfilled a similar function early on, highlighting a gender gap in our pilot version of the program. Focusing on women alone is common in reproductive health programs. In Mama SASHA, men had to be involved in household decisions on land use and influence food selection and preparation choices for family consumption.

Our use of the impact pathway had a number of benefits: 1) clarifying the important contribution of activities in each sector to overall impacts of mutual interest; 2) making visible to partners the linkages across and between outputs along causal pathways, fostering careful specification of monitoring indicators; 3) helping ensure parsimonious data collection by focusing on key variables in our program theory; 4) highlighting more difficult to document qualitative changes which underpinned success; and 5) providing a focus across disciplines to leverage integration (Klein, 2008). However, we noted several areas where the linkages across sectors were vulnerable, requiring substantial time investments in joint meetings and problem solving. A key question for sustainability is how articulation between the agricultural and health services sectors can be sustained through shared governance mechanisms, despite the challenges involved (Alpha, 2013). Our work with existing farmer and social groups, and the decentralization of government functions to the county level in Kenya’s constitutional reforms, may both foster continued joint, multi-sectoral planning (IFPRI/FAO, 2013).

A key vulnerability of multi-sectoral integration also surfaced. Our program required agricultural inputs to be exactly matched in time, quantity and quality to the cycle of care in antenatal care. Sweetpotato planting materials (vines) are perishable, so they could not be provided at the point of consumption of health care. Furthermore, there is a lag between access to vines, planting and consumption of OFSP roots. Hence, a key point of articulation between two relatively separate systems (agricultural extension and maternal-child health care), and a key innovation of the Mama SASHA program, was the voucher for vine. The physical point of exchange between the two systems could be tracked in both. During months of the regular dry season in the region, women were reluctant to pick up vines that they could not keep alive, so voucher redemption rates dropped dramatically affecting both the land area planted by vine multipliers (needed to ensure a steady flow of healthy, mature vines) and Vine Multiplier income flows, as they were reimbursed for vouchers redeemed. Two adaptions were thus required. For the vine multipliers, additional investment was required to reduce risk through provision of pumps for irrigation, which they could use both on OFSP, to maintain vine availability, and for other crops, which they could sell to maintain their incomes. For the women participants, the period during which they could redeem vouchers was extended. These adaptions maintained the crucial implementation link between the agricultural and health sectors to make sure OFSP would be planted by participating women and their households.

In low income country settings, evaluation designers must also face the tradeoffs required between substantial resources invested in different evaluation components to meet different disciplinary “cultures of evidence”, and the option of using scarce resources directly for development program purposes. Building trust and joint practices across historically separate sector systems required support for travel and face to face meetings to resolve communication and operational challenges and build understanding of the complementary roles of evaluation and implementation. Among partners, an innovative partnership checkup tool was used every six to eight months that permitted each individual to assess how they felt the partnership was functioning; then a facilitator guided a discussion among the whole team about how to improve weak areas in the partnership. Many felt this tool enabled issues to be dealt with before they turned into problems.^12^

Monitoring visits to health facilities required additional forms to be able to properly track participant women, which increased documentation workload. Maintaining relationships with a control group of health facilities and villages incurred personnel costs, as did provision of interventions, even partial, at the end of the evaluation period. Mounting censuses to identify households with pregnant and lactating women, and then coming back and surveying them, two times during a three year period was resource intensive. Even more so were detailed dietary assessments and biological measures longitudinally on a sub-sample of participants. Juggling a combination of planning, design and funding applications during the implementation of an ambitious multi-sectoral intervention was a challenge for field teams of whom we expected so much. Maybe we tried too hard to “do it all” (Morell, 2010). Such considerations must be part of the discussion when planning, choosing, and implementing evaluations of multi-sectoral, intervention programs.

## 7 Lessons learned

Three key lessons for development program planners and evaluators emerged that are applicable when planning and evaluating a multi-sectoral program:

1) The opportunities for multiple areas of change afforded by multi-sectoral programs outweigh the challenges. Nevertheless, substantial time is needed for planning, pilot testing, and re-designing based on preliminary findings;
2) Thinking through the program theory and updating the impact pathway can integrate across sectors and disciplines and guide the appropriate combination of qualitative and quantitative monitoring; and
3) Multiple evaluation components may be needed to respond to the different cultures and disciplines of evidence present in different sectors, but doing more is associated with program and evaluation staff burden.

## Figures and Tables

**Table 1:** Regular meetings for planning, integration and implementation in Mama SASHA.

Type of meeting	Frequency	Participants
Stakeholder—network	Annually Dec 2009 to May 2013	Cross-sectoral stakeholders: operational partners plus district and district nutritionists and provincial representative of Ministry of Health, home economists and provincial representative of Ministry of Agriculture, and other agriculture and nutrition relevant development implementing partners^a^
Partner	Quarterly	Formal operational partners: CIP program leader & agronomist, APHIA coordinator, development NGO directors and agricultural extension officers, representative of Kenya Agriculture Research Institute
Monitoring—feedback	Monthly	Actors on the ground: PATH project officer, trained community health workers, vine multipliers, community health extension workers, ante-natal care nurses, agricultural extension officers

APHIA—AIDS, Population and Health Integrated Assistance Program; CIP—International Potato Center; PATH—formerly Program for Appropriate Technology, now acronym only.

a Development implementing partners as per Ministry of Public Health & Sanitation, Republic of Kenya, 2012.

**Table 2:** Mama SASHA evaluation components, purposes, and key expected contributions.

Component	Purpose(s)	Key expected contributions
Random allocation of program by health facility catchment area	To respond to demand for more rigorous impact evaluations	Estimates of program effectiveness bolstered by comparison with a control set of villages
Program implementation evaluation (ii)	To check on acceptability, uptake, and challenges in implementation	Qualitative evaluation of facilitators of and barriers to implementation and participation
Repeat village—household surveys pre and post program intervention	To assess changes in prevalence of target agricultural (OFSP production), nutritional (OFSP consumption) and health (maternal and infant) practices relevant to the program	Estimates of program coverage in intervention villages, and extent of change in target practices attributable to the program (controlling for co-variate prevalence) i.e. effectiveness
Longitudinal study of women-infant pairs	To determine the extent to which program related practice changes were associated with relevant health indicator changes	Linking of OFSP^a^ production and use with maternal and infant diet and health outcomes including biological measures of Vitamin A status and anemia i.e. individual-pair change
Program costing study	To document the resources required for program implementation and value them	Estimates of program costs and input to cost-effectiveness analysis to jointly inform health and agriculture policy makers

a OFSP = orange-fleshed sweetpotato.

## Footnotes

1 Abbreviations used in the paper: APHIA—AIDS, Population and Health Integrated Assistance Program (APHIA II and later APHIA plus); CIP—International Potato Center; DALYs—Disability Adjusted Life Years; SASHA—Sweetpotato Action for Security and Health in Africa; NGO—Non-Governmental Organizations; OFSP—Orange-Fleshed Sweetpotato; PATH now known by its acronym only.

2 In June 2011, Mumias sugar launched vitamin A fortified sugar. However, this fortified sugar only appeared sporadically in the market throughout the intervention period and was not in wide use. Other vitamin A fortified products exist on the market, such as Blueband margarine and infant cereals but their price was beyond the reach of average rural consumers. Any use of these fortified products was captured in the vitamin A food frequency module.

3 The two orange-fleshed sweetpotato varieties used in this project, Kabode (NASPOT 10 O) and Vita (NASPOT 9 O), were conventionally bred by the National Crop Resources Research Institute (NaCRRI) in Uganda and subsequently released officially in Kenya with CIP support. Varieties bred for use in the Lake Victoria zone must show significant resistance to sweetpotato viruses, as virus pressure is high in this area.

4 The initial design called for individual level counselling at ANC visits by the nurses and group training sessions by community health workers at pregnant women’s club meetings. However, in some of the busier facilities, nurses said they had insufficient time for individual counselling and would either conduct group sessions with the women attending the ANC day or ask the community health worker to come and counsel the women.

5 Vogus Abby, Cagley JH, Heim J, Kukathas U. Vouchers for increasing healthcare access for women and children: What is the evidence? Submitted in partial fulfillment of the requirements for the certificate of International Development Management and Policy, University of Washington. 2010, 52 pp.

6 Machira YW, Levin C, Kurji J, Ouedrago H, Kedera E, Loechl C, Cole DC, Low J. Operational research to inform the design of an integrated agriculture and health delivery strategy to improve pregnant women’s health and maximise the nutritional benefits of orange fleshed sweetpotato in Western Kenya. Kemri-CDC 2nd Nyanza Scientific Conference, Tom Mboya Labour College, Kisumu, Kenya. 19 January 2012.

7 Machira Y, Levn C, Grant F, Kedera E, Wamalwa M, Bingham A, Cole DC, Low J. Feasibility And Acceptability Of Integrating Vitamin A Rich Orange Fleshed Sweetpotato Into Antenatal Care Services In Western Kenya. Poster 803, International Union Of Nutritional Sciences–International Congress Of Nutrition, Granada Spain 15–20 September 2013.

8 Grant F, Webb Girard A, Low J, Cole DC, Levin C, Kariuki S, Sindi K. Understanding current sweetpotato utilization and nutritional status of groups vulnerable to vitamin A deficiency in Western Kenya; an endline assessment. Protocol approved by KEMRI (Kenyan Medical Research Institute) October 2013.

9 Grant F, Webb Girard A, Kariuki S, Mahungu S, Cole DC, Levin C, Low J. Cohort study of the impact of the Mama Sasha project—an integrated agriculture and health intervention to maximise the benefits of Orange Fleshed Sweetpotato on the Vitamin A and health status of mothers and their infants from pregnancy through 9 months postpartum. Protocol approved by KEMRI (Kenyan Medical Research Institute), 15 October 2012.

10 HarvestPlus. ‘Reaching and Engaging End Users (REU) with Orange Fleshed Sweet Potato (OFSP) in East and Southern Africa. Final Report submitted to the Bill and Melinda Gates Foundation. July 9, 2010.

11 Levin C, Self J, The Mama SASHA Study Team (******* Ellah Kedera, Frederick Grant, Amy Webb Girard, Donald Cole, Jan Low). Financial costs of Mama SASHA: A project to improve health and nutrition through an integrated orange flesh sweetpotato production and health service delivery model. Report to PATH, Kenya & Seattle. April 2014. 38 pp & Appendices.

12 Report of Partners Planning Meeting on 10th–11th June 2013 at Greenvalle Hotel, including Item 8 On Partners’ Health Checkup.
